# Self-assembling ferritin nanoplatform for the development of infectious hematopoietic necrosis virus vaccine

**DOI:** 10.3389/fimmu.2024.1346512

**Published:** 2024-01-29

**Authors:** Sohrab Ahmadivand, Zeljka Krpetic, Merce Márquez Martínez, Marlid Garcia-Ordoñez, Nerea Roher, Dušan Palić

**Affiliations:** ^1^ Faculty of Veterinary Medicine, Ludwig-Maximilians University Munich, Munich, Germany; ^2^ Biomedical Research Centre, School of Science Engineering and Environment, University of Salford, Salford, United Kingdom; ^3^ Institute of Biotechnology and Biomedicine (IBB), Universitat Autònoma de Barcelona, Barcelona, Spain; ^4^ CIBER de Bioingeniería Biomateriales y Nanomedicina (CIBER-BBN), Barcelona, Spain

**Keywords:** ferritin nanoparticles, IHNV, self-assembling vaccine, fish viruses, ZFL cells, macrophages, protein stability

## Abstract

Self-assembling protein nanoparticles are used as a novel vaccine design platform to improve the stability and immunogenicity of safe subunit vaccines, while providing broader protection against viral infections. Infectious Hematopoietic Necrosis virus (IHNV) is the causative agent of the WOAH-listed IHN diseases for which there are currently no therapeutic treatments and no globally available commercial vaccine. In this study, by genetically fusing the virus glycoprotein to the H. pylori ferritin as a scaffold, we constructed a self-assembling IHNV nanovaccine (FerritVac). Despite the introduction of an exogenous fragment, the FerritVac NPs show excellent stability same as Ferritin NPs under different storage, pH, and temperature conditions, mimicking the harsh gastrointestinal condition of the virus main host (trout). MTT viability assays showed no cytotoxicity of FerritVac or Ferritin NPs in zebrafish cell culture (ZFL cells) incubated with different doses of up to 100 µg/mL for 14 hours. FerritVac NPs also upregulated expression of innate antiviral immunity, IHNV, and other fish rhabdovirus infection gene markers (mx, vig1, ifit5, and isg-15) in the macrophage cells of the host. In this study, we demonstrate the development of a soluble recombinant glycoprotein of IHNV in the E. coli system using the ferritin self-assembling nanoplatform, as a biocompatible, stable, and effective foundation to rescue and produce soluble protein and enable oral administration and antiviral induction for development of a complete IHNV vaccine. This self-assembling protein nanocages as novel vaccine approach offers significant commercial potential for non-mammalian and enveloped viruses.

## 1 Introduction

Vaccination strategies to prevent and control viral pathogens and disease outbreaks in aquaculture need to be optimized for efficacy and stress-free administration, with consideration of production and delivery costs, environmental risks, and regulatory compliance (1, 2). The traditional vaccine production and application approach is based on the use of inactivated or attenuated viral vaccines, which are commercially available for some viral diseases (3). Even with the relatively low risk of possible reversion to virulence and spreading of vaccine strain in the environment, such vaccines still offer benefits including induction of a strong immune response when administered via stressful intraperitoneal injection and combined with oil adjuvants (1–3). DNA-based vaccines have recently shown promising results against certain viruses, but they raise safety issues regarding genetically modified organisms (GMO) and, despite the efficacy of some oral formulations, are administered by labor-intensive intramuscular (i.m) injection (4–6). Novartis’ APEX-IHN^®^ DNA vaccine is an example, approved for i.m use in Atlantic salmon in Canada, but not elsewhere due to GMO safety concerns (4).

Another vaccine approach is the development of subunit vaccines, including the production of viral antigen proteins in a heterologous expression system. Although safer and more cost-effective to produce, their efficacy is variable and the development of oral subunit vaccines remains challenging due to gastrointestinal barriers (7). Most subunit vaccines require intraperitoneal (IP) injection, which is expensive and difficult to administer to young fish and causes stress and injury to the fish (1).

Infectious hematopoietic necrosis virus (IHNV), the enveloped (-) ss RNA virus of the Rhabdoviridae family, is the major cause of significant losses in the global trout and salmon aquaculture industry, with mortality rates reaching 90%, mainly in small fish. Neither therapeutic treatment nor a safe/mass delivery global commercial IHNV vaccine is currently available (8).

Research on vaccine development against IHNV has been ongoing for over four decades and several vaccine candidates based on inactivated virus, recombinant G protein expressed from prokaryotic and eukaryotic systems, attenuated virus strains, and DNA vectors have been shown to be efficient in eliciting specific and long-lasting protective immunity in fish but none has met all the requirements for commercialization, especially safety and mass delivery to a large number of highly susceptible small fish (9).

Approaches including the production of a recombinant IHNV glycoprotein in *E. coli* (10), yeast (11), and baculovirus/insect cells (12), failed to elicit high levels of protection against homologous virus challenge, although in some cases, neutralizing antibodies were detected in the sera of immunized fish. Furthermore, these low levels of protection were achieved only when recombinant glycoprotein was administered by intraperitoneal injection in combination with an adjuvant (13). Similarly, synthetic peptides representing putative antigenic determinants of the IHNV glycoprotein were poorly immunogenic (14).

Glycoprotein G is the unique target of neutralizing and protective antibodies, and recombinant subunit vaccines have been based on this protein (9–14). However, the complexity of expression and the challenge of producing soluble and functional recombinant glycoprotein to induce protective immunity, as well as the route of administration due to the protein stability issues, remain the main obstacles to the development of an effective IHNV subunit vaccine for mass delivery to fish fry (1, 2).

Displaying structurally defined antigenic epitopes in high copy numbers on the surface of self-assembling nanoparticles (NPs), such as VLPs or protein nanocages, to improve antigen stability and immunogenicity, with targeted delivery and slow release, is one of the novel technologies to address the challenges of subunit vaccines (15).

Although no VLP-based vaccines have yet been licensed for aquaculture, there is considerable experimental evidence of the potential of this type of vaccine for protecting fish against viral diseases such as infectious pancreatic necrosis (IPN) (16), pancreatic disease (PD) (17), and viral nervous necrosis (VNN) (18).

However, in contrast to VLPs of the non-enveloped viruses assembly process with only capsid proteins, enveloped viruses require an additional membrane component for assembly into mature virions, leading to enveloped VLPs not being structurally uniform and therefore difficult to characterize (19). A possible solution for the presentation of target antigens would be to display them to the host on the surfaces of self-assembled protein nanocages, which, in lieu of lipid membranes and matrix proteins, serve as an ideal scaffold for the enveloped viruses such as IHNV.

Ferritin is a major intracellular iron storage protein present in most living organisms, with 24 identical subunits that spontaneously self-assemble and form NPs complexes (20). Ferritin nanocages can enhance the immunogenicity of antigens by displaying them on their outer surface in an orderly manner, similar to the whole organism vaccines, leading to long-lasting immunity, which recently has been proven to work well both *in vitro* and *in vivo* for some mammalian viruses (21–25). The vaccines have been developed using eukaryotic host cells such as human embryonic kidney cells, and prokaryotes (*E. coli*) expression systems, delivered successfully through different routes in animal models (21–25). The use of ferritin NPs as an antigen scaffold can therefore improve the immunogenicity of subunit vaccines, and can also be considered as a promising platform for oral vaccine development due to its unique structure and stability under harsh temperature and pH conditions (20, 26).

This study describes for the first time the use of the self-assembling ferritin nanocages as vaccine platform for a non-mammalian virus. This approach resulted in the successful development of a soluble IHNV glycoprotein on a self-assembled ferritin nanoparticle in a low-cost production system (*E. coli*), which is biocompatible, stable, and effective for antiviral induction.

## 2 Materials and methods

### 2.1 Cell cultures

Zebrafish liver (ZFL) cells (CRL-2643, ATCC) were cultured in DMEM 4.5 g/mL glucose (Gibco) supplemented with 0.01 mg/mL insulin, 50 ng/mL epidermal growth factor, 10% (v/v) heat-inactivated FBS, 5% (v/v) antibiotic/antimycotic, and 0.5% (v/v) heat-inactivated trout serum at 28°C with 5% CO_2_ as previously described (27). Adherent rainbow trout head kidney macrophages (RT-HKM) were isolated from the head kidney as described previously (27, 28), and cultured in the same supplemented medium at 15°C with 5% CO_2_. After 24 h, the cells were washed in PBS to remove nonadherent cells, and the media was replaced with fresh culture media and then changed every 48 hours for up to 5 days, which were used for immune gene expression assays.

### 2.2 Vector construction and cloning

The glycoprotein (G) gene fragment (184 aa) (29), from the ectodomain-encoded region of the European standard isolate of IHNV (X89213) fused (GGSSRSS linker) to N-terminal of *Helicobacter pylori* ferritin (WP_000949190) and a His-tag in the C-terminus. Then the construct was synthesized by Invitrogen GeneArt Gene Synthesis (ThermoFisher, DE) and cloned using the pET22b vector with XhoI and BamHI sites in *E. coli* (DH5α) bacteria. The IHNV-G fragment and ferritin control constructs and red fluorescent protein (iRFP-His) non-immunological control protein were similarly designed and cloned (27).

### 2.3 Protein expression and purification

For protein expression, vector constructs were isolated using the QIAprep Spin Miniprep Kit (Qiagen, USA) and transformed to BL21 *E. coli* strain with XhoI and BamHI enzyme digestion verification. Expression then was performed using BL21(DE3) grown in LB with 100 μg/mL ampicillin and induced with IPTG at 1 mmol/L. The expression temperature was set at 20°C for overnight cultivation. Cells were harvested by centrifugation (5000 g for 15 min at 4°C) and stored at -80°C. Protein expression was confirmed using SDS-PAGE and Western blot using an anti-His-tag antibody. Despite the different expression and purification conditions, the IHNV-G fragment was an insoluble protein (**Supplementary Figure 1**), while the FerritVac was soluble. After cell lysis, the recombinant proteins were purified by immobilized metal affinity chromatography (IMAC) on an ÄKTA Pure FPLC system. Proteins were eluted with a linear gradient of elution buffer (20 mM Tris HCl (pH 8.0), 50 mM NaCl, 500 mM imidazole) after selective binding to a HisTrap HP 1 mL column (GE Healthcare). Selected protein fractions were dialyzed to remove imidazole in the 20 mM Tris HCl (pH 8.0), 50 mM NaCl buffer. The Ferritin and FerritVac proteins were then also submitted to size exclusion chromatography (SEC) in a HiLoad 16/600 Superdex 200 pg column (Cytiva, Marlborough, MA, USA, ref. GE28-9893-35) with isocratic elution in 20 mM TRIS-HCl pH 8.0. Proteins with different sizes were eluted by monitoring their absorbance at 280 nm. The peaks obtained were compared with known molecular weight marker proteins (GE-Healthcare, USA). Fractions corresponding to monomers and trimers of the proteins were collected separately (**Supplementary Figure 2**). The final protein concentration was determined using a NanoDrop and Qubit™ Protein Broad Range (BR) Assay Kits (ThermoFisher, DE).

### 2.4 SDS-PAGE and western blotting

The SDS-polyacrylamide gel electrophoresis (SDS-PAGE) of the proteins was carried out on a 5-12% Bis-Tris gel with Coomassie blue visualization. For Western blotting, briefly after separation by SDS-PAGE, the proteins were electrophoretically transferred to PVDF membranes (Millipore) and then incubated in TTBS containing 10% skim milk for 1 h at room temperature to block non-specific binding during incubation with antibodies. The membranes were incubated overnight at 4°C with 1:10,000 anti-His primary antibody (ThermoFisher, DE) in 20 mL TTBS containing 10% skim milk, washed three times for 10 minutes at 25°C with 0.01% TTBS, and incubated for 1 hour at room temperature with 1:10,000 goat anti-mouse secondary antibody (ThermoFisher, DE) in the same buffer. Then, chemiluminescence visualization was performed using the SuperSignal™ Western Blot Kit (ThermoFisher, DE) and a BioRad ChemiDoc.

### 2.5 Dynamic light scattering

Dynamic light scattering (DLS) using a Zetasizer Nano ZS (Malvern Instruments, UK) was used to determine the particle size distribution and zeta potential of the protein NPs at 25°C, confirming the colloidal stability. Triplicate measurements were performed on 100 μL of each sample. Prism 9 (GraphPad software) was used to generate volume size distribution (nm) histograms.

### 2.6 Transmission electron microscopy

For transmission electron microscopy images, 10 μL of the protein samples (Ferritin and FerritVac NPs) were applied to carbon-coated copper grids for 10 min. The grids then were negatively stained with 10 μL of 2% (w/v) uranyl acetate solution and wiped out with filter paper strips after 1 min. The resulting grids were visualized with a JEOL 1400 (JEOL Ltd.) TEM instrument at 120 kV, and images were captured with a CCD GATAN ES1000W Erlangshen camera (Gatan Inc.). Size measurements were performed using ImageJ software (U.S. National Institutes of Health, Bethesda, MD), averaging 50 individual measurements for each protein nanoparticle.

### 2.7 Stability assays

The stability of FerritVac and ferritin NPs in 100 µL 20 mM Tris-HCl and 50 mM NaCl buffer (pH: 8.0) was measured by DLS under different storage conditions of 4°C and -80°C freeze-thaw after 2 weeks and under the same conditions found in the gastrointestinal tract of trout (pH:8.0 and pH:3.0) at the optimum temperature for trout fry culture and IHNV infection (15°C) for 2 and 4 h (8, 30). The pH of the buffer was adjusted with HCl and then exchanged using Pierce Protein Concentrator columns (ThermoFisher, DE).

### 2.8 Cytotoxicity assay with ZFL cell line

The MTT assay according to Thwaite et al. (31) was used to determine the cytotoxic effects of NPs on ZFL cells. After 2.5 h on minimal media, cultures were stimulated with NPs at 1, 5, 25, 50, and 100 µg/ml in duplicate wells and plates, and incubated for 14 h at 28°C. Cultures were washed in PBS and 10% MTT substrate (Sigma-Aldrich) was added. Cells without NPs were used as controls. The cells were then further incubated at 28°C for 30 min. The solution was removed and solubilized in DMSO, and the absorbance was read at 550 nm on a Victor 3 plate reader (PerkinElmer). The experiment was repeated three times. Data normalized to control readings set at 100% and analyzed by one-way analysis of variance (ANOVA).

### 2.9 Expression of antiviral genes in RT-HKM primary cells

The trout head kidney macrophage primary cells were simulated with the different doses of FerritVac NPs (10, 25, and 50 µg/mL) for 14 hours at 15°C, and compared with untreated cells and cells incubated with 25 µg/mL of ferritin and iRFP proteins as controls in triplicate plates. Total RNA was then extracted from the cells using the ReliaPrep™ RNA Cell Miniprep System (Promega), and cDNA was synthesized using the GoScript™ Reverse Transcription System Kit (Promega). The qPCR was performed using Fast SYBR™ Green Master Mix (ThermoFisher, DE) (32),, for gene markers of the innate immune response to viral infection (*mx, vig1, ifit5*, and *isg-15*) (33, 34),. The expression of the target genes has been corrected based on a reference gene (*EF-1 α*) and calculated relative to the control via the 2^- ΔΔCt^ method (35). Primer details are shown in the **Supplementary Table 1**.

### 2.10 Statistical analysis

Analyses were performed using SPSS 23 and GraphPad Prism 9 software. A one-way ANOVA was performed with the Duncan test to compare all group means and Dunnett’s multiple comparisons test between each treatment and control mean, at a significance level of *p <*0.05.

## 3 Results

### 3.1 Purification and characterization of NPs

**Figure 1:**
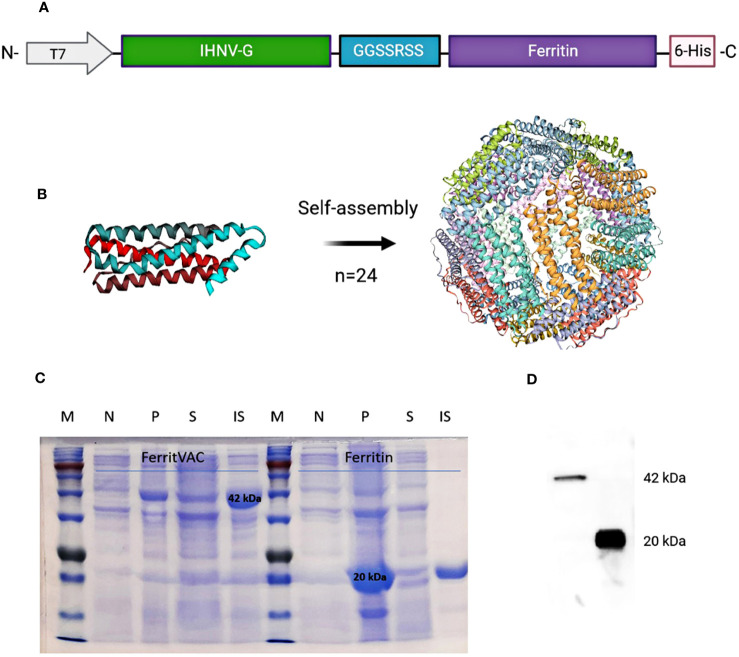
Construction and production of IHNV vaccine (FerritVac) using a Ferritin nanocage. **(A)** Construction of the FerritVac fusion protein: The IHNV glycoprotein fragment (aa 270 to 453) protein was linked (GGSSRSS linker) to the N-terminal of the (*H*) *pylori* ferritin with a His-tag in the C-terminus. **(B)** 3D schematic diagram of the ferritin platform. **(C)** SDS-PAGE (12%) of Ferritin and FerritVac protein NPs: Total protein production before (N) and after (P) IPTG induction for the same volume of culture sample. Soluble (S) and insoluble (IS) fractions after IPTG induction (overnight at 20°C) and cell lysis. **(D)** Western blot analysis of FerritVac (42 kDa) and Ferritin (20 kDa).

For the construction of the nanovaccine, the most immunogenic fragment in the ectodomain of IHNV glycoprotein (29) was linked to the N-terminus of ferritin (**Figure 1A**), and a nanoparticle displaying the IHNV-G (FerritVac) was generated after the self-assembly of the ferritin. **Figure 1B** shows a schematic of the FerritVac NPs. The proteins were expressed in *E. coli* and purified by Ni-chelating affinity chromatography. Purified proteins were dialyzed for imidazole removal. While the IHNV glycoprotein alone was insoluble (**Supplementary Figure 1**), we successfully expressed and purified a soluble ferritin containing the IHNV glycoprotein fragment in the *E. coli* system we successfully expressed and purified a soluble ferritin containing the IHNV glycoprotein fragment in the E. coli system (**Figures 1C** and **1D**). SDS-PAGE revealed 42, 22, 20, and 36 kDa for ferritin, IHNV-G, and iRFP, respectively (**Supplementary Figure 1**).

**Figure 2:**
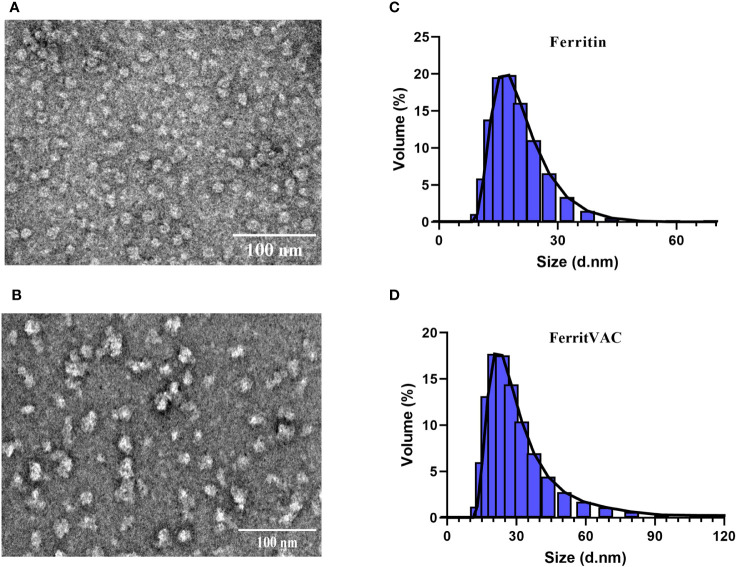
Morphological characterization and particle size distribution of Ferritin and FerritVac by TEM and DLS. Transmission electron micrographs (scale bar =100 nm) of negatively stained Ferritin **(A)** and FerritVac **(B)**. The. Size distribution of Ferritin **(C)** and FerritVac **(D)** characterized by DLS. Size distribution analysis of FerritVac nanoparticles *via* DLS showed an average diameter of 20.53 nm, which was larger than the diameter of Ferritin cages (15.8 nm).

After purification, the ferritin and FerritVac proteins were also submitted to size exclusion chromatography (SEC) to differentially elute proteins according to their hydrodynamic volumes. The fractions belonging to peaks 1 and 2 were separately collected and evaluated to remove aggregates and other low molecule impurities (**Supplementary Figure 2**). Full physicochemical characterization of the protein nanoparticles including stability in buffer solution was performed with Dynamic Light Scattering (DLS) and the morphology of the particles was determined using Transmission Electron Microscopy (TEM). The nanoparticles characterization by TEM is shown in **Figures 2A, B** and dynamic light scattering (DLS) analysis is shown in **Figures 2C, D**.

TEM micrographs show spherical Ferritin and FerritVac NPs with a narrow particle size and mean diameters of 15.8 ± 0.52 nm and 20.53 ± 1.13 nm, respectively. DLS analysis revealed a highly monodisperse population of FerritVac NPs with a low polydispersity index (0.29 ± 0.01). The resulting FerriVac NPs resulted in a larger hydrodynamic diameter, compared to ferritin, as expected. There was no significant difference between the diameter of the particles visualized by TEM and the diameter determined by DLS (**Table 1**).

**Table 1:** ζ-potential and particle size of purified FerritVac and Ferritin NPs determined by DLS and TEM.

NPs *^a^*	TEM (nm)	DLS (nm)	PDI	ζ-potential (mV)
Ferritin	16.23 ± 1.34	15.8± 0.52	0.47 ± 0.00	- 20.46 ± 0.16
FerritVac	20.73 ± 2.28	20.53 ± 1.13	0.29 ± 0.01	- 18.42 ± 1.04

^*a*^The diameter of 50 particles visualized by TEM was determined using ImageJ software [mean ± standard deviation (SD)], and DLS mean size values ± SD were obtained by considering the volume-based distribution. PDI, polydispersity index.

Zeta potential (ζ-potential) measurements confirmed the colloidal stability of analyzed dispersions (**Table 1**) with the electric charges of ferritin and FerritVac negatively charged. The ζ-potential for FerritVac NPs was -18.42 ± 1.04, similar to that of Ferritin (-20.46 ± 0.16 mV), indicating a lack of particle aggregation in dispersion.

### 3.2 The stability of NPs

**Figure 3:**
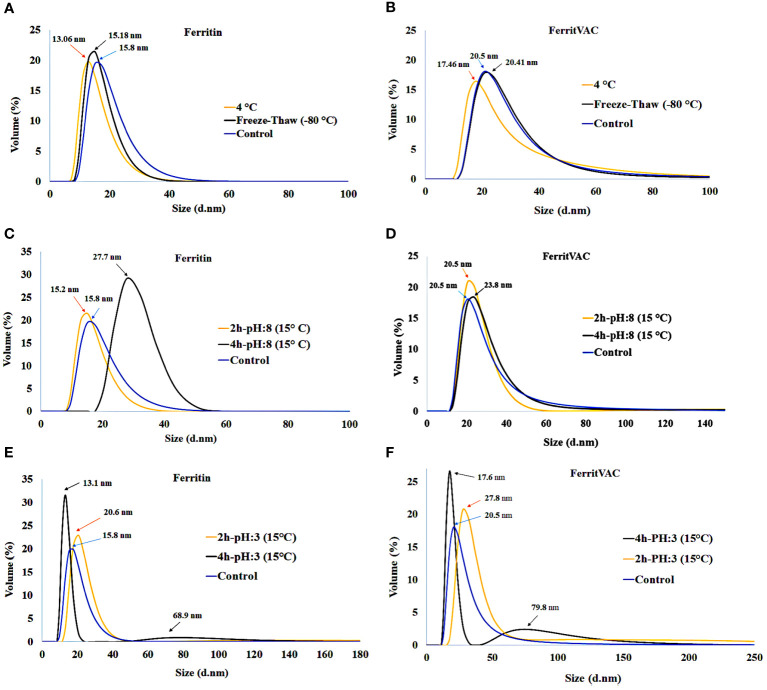
Stability of FerritVac and Ferritin NPs assessed by DLS under different storage conditions: 4°C and -80°C freeze-thaw after 2 weeks **(A, B)**, and for 2 h and 4 h at pH:8.0 **(C, D)** and pH:3.0 **(E, F)** at 15°C, which resemble the gastrointestinal pH conditions of trout, the main host of IHNV, at the optimal water temperature for the culture of the species and other salmonids. The data show a high stability of both NPs. However, over time the harsh conditions can gradually lead to disassembly and aggregation, which can be seen as a slight decrease and increase in peak size, respectively.

The stability of FerritVac and Ferritin NPs under different storage conditions was evaluated by DLS (**Figures 3A, B**). After 2 weeks of storage at 4°C and a freeze-thawing at -80°C, the protein NPs were stable and appeared as a single peak with no significant change in the size of NPs, demonstrating the monodispersity of NP samples under defined storage conditions. However, a slight decrease in particle size was observed at 4°C, which may indicate that proteins begin to disassemble over time. While freezing and thawing stress can cause proteins to denature and aggregate, this Ferritin platform demonstrated stability.

To verify the suitability for oral administration, we evaluated the stability of the NPs at pH:8.0 and pH:3.0 at 15°C, which resemble the gastrointestinal pH conditions of trout at the optimal water temperature for the culture of the species and other salmonids (**Figures 3C-F**). At pH:8.0 and incubation at 15°C of the samples, DLS analysis showed stable protein NPs after 2 h in solution and a slight increase in particle size of Ferritin NPs after 4h, indicating that the samples began to aggregate. Despite the stability after 2 h, a gradual increase in size and appearance of an aggregation peak with size of 68.9 ± 3.25 and 79.8 ± 2.63 for Ferritin and FerritVac NPs, respectively, were observed after 4 h of incubation at lower pH:3 and temperature of 15°C. Overall, the FerritVac NPs show excellent stability as Ferritin, displaying a similar environmental tolerance pattern.

### 3.3 Cytotoxicity in ZFL cells

**Figure 4:**
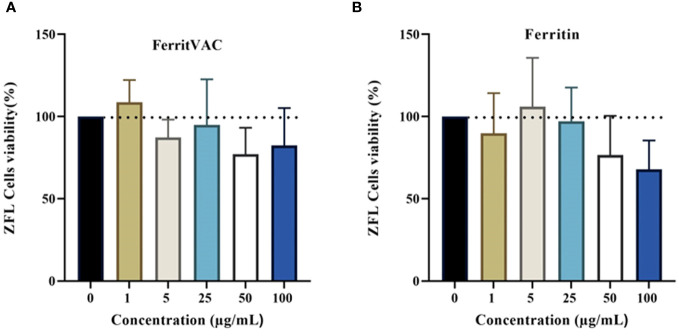
MTT assay cytotoxicity of FerritVac and Ferritin NPs in zebrafish liver (ZFL) cells: ZFL were incubated with FerritVac **(A)** and Ferritin **(B)** NPs at 1, 5, 25, 50, 100 µg/mL in duplicate wells and plates (n=4) for 14 h at 28 °C. ZFL cells without NPs incubated were used as control. After MTT treatment, incubation for 30 min, and subsequent solubilization in DMSO, absorbance was measured at 550 nm and data normalized to control readings set at 100%. A one-way analysis of variance (ANOVA) with Dunnett’s multiple comparison test was performed between each treatment and control at a significance level of *p*<0.05. None of the treatment groups were significantly different from the control (*p >*0.05).

There was no significant difference in survival between the control and any of the treatment groups in the MTT assays in ZFL cells incubated with 1, 5, 25, 50, and 100 µg/mL of each NPs for 14 h, indicating that neither FerritVac nor Ferritin NPs are cytotoxic at these doses (**Figure 4**). Cell viability values remained above 95% for both FerritVac and Ferritin up to 25 μg/mL after 14 h of exposure. However, a slight decrease with no significant difference was observed in the ZFL cells from 50 μg/mL and above, dropping to about 82% (*p* =0.25) and 70% (*p* =0.08) viability at the highest concentration (100 μg/mL) of FerritVac and Ferritin NPs, respectively.

### 3.4 Expression of antiviral genes in RT-HKM primary cells

**Figure 5:**
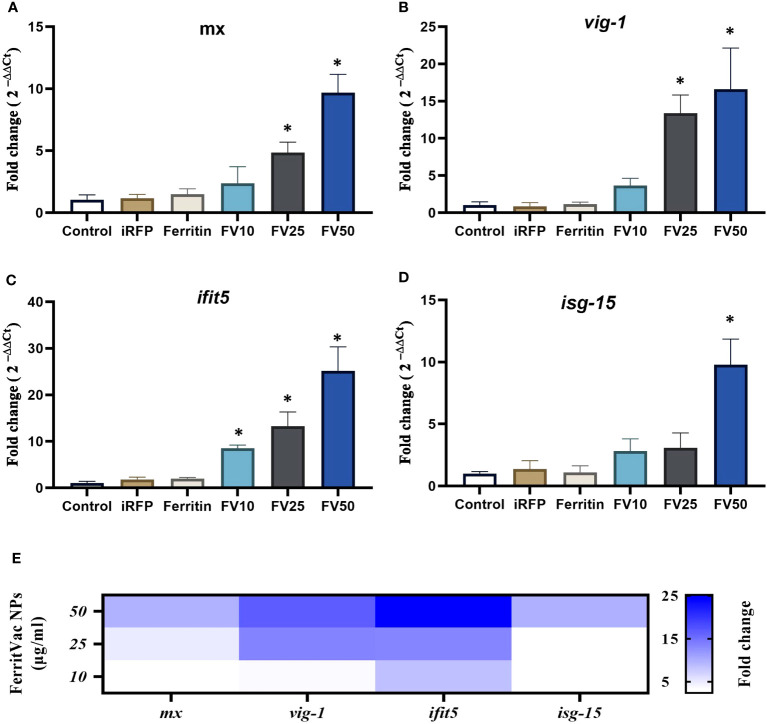
Expression of antiviral genes in trout head kidney macrophage primary cells (RT-HKM) incubated with FerritVac and Ferritin NPs. **(A)** *mx*, **(B)** *vig1*, **(C)** *ifit5*, and **(D)** *isg-15* gene expression in the cells incubated for 14 h as follows: unstimulated cells (control), iRFP (25 µg/mL) as an immunogenically irrelevant control, Ferritin (25 µg/mL) and different concentrations of FerritVac NPs 10 µg/mL (FV10), 25 µg/mL (FV25) and 50 µg/mL (FV50). **(E)** Heat map comparing expression changes of analyzed genes in RT-HKM cells incubated with FerritVac NPs. Samples are from three independent experiment plates from three different fish. Data were normalized based on the endogenous *EF-1a* gene and presented as mean fold change relative to unstimulated control cells; 2^-ΔΔCT^ method). A one-way ANOVA was performed with Duncan to compare all groups’ means, and Dunnett’s multiple comparisons test between each treatment and control mean, at a significance level of *p <*0.05. Asterisks indicate a significant difference compared to the control (* *p* < 0.05).

To investigate the ability of FerritVac NPs to stimulate the expression of innate antiviral genes in antigen-presenting immune cells, we treated the rainbow trout head kidney macrophage primary cells with the NPs. Untreated cells and cells treated with 25 µg/mL of Ferritin and iRFP were used as controls. Genes tested included *mx, vig1, ifit5*, and *isg-15*, which are relevant markers of innate antiviral immunity, IHNV, and other fish rhabdovirus infections (33, 34). For all genes tested, FerritVac NPs exposure induced up-regulation significantly different from the controls in a dose-dependent manner (**Figure 5**).

The qPCR analysis revealed a significant increase in the expression of *mx* and *vig1* genes of macrophage cells incubated with 25 or 50 µg/ml dose of FerritVac NPs compared to the control groups as well as the lower dose of 10 µg/ml of NPs (**Figures 5A, B**). While the *ifit5* gene was significantly up-regulated in all dose groups of FerritVac NPs (**Figure 5C**), the *isg-15* gene was only significantly up-regulated (about 10-fold) in the cells treated with the higher dose of 50 µg/ml of NPs (**Figure 5D**). No significant differences were observed between cells incubated with 25µg/ml iRFP or ferritin NPs compared to untreated controls (*p >*0.05).

## 4 Discussion

Viral diseases in aquaculture are difficult to control due to the lack of approved and affordable antiviral veterinary medical products, as well as challenges in developing effective and safe viral vaccines that can be mass-administered to young fish, especially during early stages of their life cycle characterized with high susceptibility to virus infections (1–3). Gaps in treatment and prevention of viral diseases are associated with periodic disease outbreaks, animal welfare problems, and losses in aquaculture worldwide, threatening the long-term sustainability of the sector (36–38).

Self-assembling protein nanoparticles, such as ferritin, have shown remarkable thermal and pH stability, monodispersity, small uniform size, biodegradable, biocompatible, cost-effective mass production, reversible spontaneous assembly/disassembly, and surface conjugation by genetic or chemical means, addressing the issue of stability and immunogenicity of safe subunit vaccines and providing broader protection against viral infections even through oral administration (20, 26, 38).

*Helicobacter pylori* self-assembling ferritin has been used for the development of safe subunit nano vaccines, against some human and other mammalian viruses such as Middle East respiratory syndrome-coronavirus (MERS-CoV) (19), Influenza A virus (21), HIV (22), rotavirus A (23), SARS-CoV-2 (24), Canine distemper virus (25), classical swine fever virus (39), foot-and-mouth disease virus (40), hepatitis C virus (41), and Zika Virus (42), which have been produced in bacteria, insects and mammalian cells systems. In this study, we have for the first time used self-assembling ferritin nanocages as a vaccine platform against a non-mammalian viral pathogen (IHNV).

It has been reported that multiple engineered nanoparticle-based vaccines using *E. coli* expression system do not support soluble protein purification and fail to assemble as intended (15). While the IHNV glycoprotein alone was insoluble, we successfully expressed and purified soluble ferritin NPs containing the IHNV glycoprotein fragment in the low-cost *E. coli* production system. This suggests that a ferritin nanocage can be used as a novel vehicle for the rescue and production of soluble proteins that are otherwise difficult to obtain using conventional methods such as the prokaryote system.

The presence of spherical NPs observed under TEM confirmed that the FerritVac (IHNV glycoprotein-Ferritin) monomer that was produced in bacterial cells, maintains its self-assembling properties and is able to form the expected nanostructure. Moreover, the NP size determined by electron microscopy (around 20.73 ± 2.28 nm) was in accordance with the DLS results measurements, and with previously characterized *H. pylori* ferritin-based vaccine candidates, yielding nanoparticles with 20-40 nm diameter after self-assembly (22, 23, 43, 44), the size range ideal for cellular uptake and B-cell activation (45).

The observed increase in diameter of FerritVac compared to that of ferritin alone could be attributed to the presence of the exogenous protein, which may reflect the modification and size of NP cages observed in this study.

We also measured the ζ-potential of Ferritin and FerritVac NPs, indicating negative values and within the range recently reported for a ferritin-based vaccine against the Zika virus by Rong et al. (42). Zeta potential measurements refer to the potential difference between the dispersion medium and the stationary layer of the fluid surrounding the dispersed particle, including the potential difference between the particle surface and surrounding liquid medium, representing the measure of colloidal stability of nanoparticles in dispersion (46). In our study, the display of IHNV glycoprotein on the Ferritin cage did not affect the stability of the nanoparticles.

The Ferritin protein complex has remarkable thermal and pH stability, withstanding temperatures of up to 80 - 100°C (20, 47). In this study, both the protein FerritVac and the ferritin NPs showed no significant changes after 2 weeks of storage at 4°C and a -80°C freeze-thaw cycle. It is noteworthy that these NPs were stored at 4°C for approximately one week during the process of purification, dialysis, and SEC prior to the stability tests, which indicates an even longer stability of the NPs. We also determined the stability of the NPs at pH:8.0 and pH:3.0 at 15°C, mimicking the pH conditions in the rainbow trout gastrointestinal (GI) tract and optimal water temperatures for the culture of this species and other salmonids in an effort to indicate suitability for oral administration of FerritVac to the rainbow trout juveniles at the age susceptible to IHNV (<6 months) (48). The pH of different sections of the rainbow trout GI tract after feeding remains acidic (pH 3.5) for at least 2h (30). However, in our study, both FerritVac and ferritin showed signs of degradation only at time points after 4 h in the low pH (3.0) conditions, as these conditions gradually lead to disassembly and aggregation, evidenced by the decrease and increase in the NP size, respectively. Similarly, it has been shown that the Ferritin protein shell begins to disassemble at pH below 3.4, but this process is reversible by subsequent elevation to higher pH values, making the platform suitable for antigenic protein delivery as well (26, 49).

The biocompatibility of active protein-based materials is critical for their biomedical use, therefore, zebrafish liver (ZFL) cells were used as a model to investigate the cytotoxicity of the Ferritin-based nanoparticles, which have already been used for some fish viral proteins (27, 31). The results of the MTT assay showed that neither FerritVac nor ferritin NPs significantly reduced ZFL cell viability compared to the control group at any of the concentrations tested after 14 hours of incubation, suggesting that these spherical nanostructures are biocompatible with living cells in an *in vitro* system. Considering zebrafish are a widely accepted biomedical research animal model, the importance of liver hepatocytes in detoxification processes, and no relevant previous studies regarding ferritin/ferritin-protein complex toxicity in this model system, our findings have significance not only for aquatic vertebrates but also for mammalian, including human being, studies.

Our results showed that FerritVac NPs upregulated the expression of innate antiviral and relevant gene markers of IHNV and other fish Rhabdovirus infection, including *mx, vig1, ifit5*, and *isg-15*, in antigen-presenting cells, trout head kidney macrophages (33, 34). Type I IFNs interfere with viral infection through the induction of a vast repertoire of ISGs via the Jak/STAT pathway, some of the ISGs exerting a direct antiviral activity such as mx, viperin/vig1, and isg15 (50), the expression of which can be simulated during IHNV infection (34). While ISGs are intrinsically located downstream of IFN in the antiviral pathways induced by viral infections, a number of them, such as viperin/vig1, are able to up-regulate type I IFNs and are therefore involved in positive feedback regulatory loops (50–52). FerittVac NPs from 25 µg/ml and above induced upregulation of *mx* and *vig1* genes in RT-HKM cells, however, *isg-15* was upregulated only at the highest dose of the NPs. Previous studies showed that over-expression of ISG15 in EPC cells is sufficient to induce antiviral activity against Novirhabdoviruses (IHNV, VHSV), Iridoviruses (EHNV) or Birnaviruses (IPNV) (50),. ISGylation, which targets cellular proteins such as TRIM25 and viral proteins such as the P and NV of IHNV, is required for the inhibition of the virus (52). Further, interferon-induced proteins with tetratricopeptide repeats (IFITs) bind to and regulate the functions of cellular and viral RNAs and proteins, thereby inhibiting viral replication (53). FerritVac NPs upregulated the ifit5 gene at all incubated doses. The ifit5 can be upregulated by type I interferons (IFNs) like IFITs, and the dependency of *ifit5* expression on activation of the Jak/STAT pathway has also been confirmed (54). Since there was no gene induction in the RT-HKM cells incubated with the ferritin-only NPs, these results indicate that immunogenicity and antiviral activity of the IHNV-G fragment was developed via the ferritin platform, most likely through the type I IFN-mediated Jak/STAT signaling pathway.

In conclusion, our study, for the first time, describes the use of the ferritin self-assembling nanocages as a vaccine platform for a non-mammalian viral pathogen. This approach resulted in the successful development of a soluble IHNV glycoprotein on a self-assembled ferritin nanoparticle, combining the advantages of glycoprotein antigen and ferritin nanoplatform to develop an effective and safe oral IHNV vaccine. Here, we also provided an initial dataset about the self-assembling protein nanocages approach with the potential to develop into a novel vaccine platform to be used in the prevention and control of aquaculture viral diseases. Further studies are needed to evaluate adaptive immune responses, safety, and efficacy of the FerritVac *in vivo* and to evaluate the applicability of this platform for other viruses.

## Data availability statement

The original contributions presented in the study are included in the article/**Supplementary Materials**, further inquiries can be directed to the corresponding author/s.

## Ethics statement

Ethical approval was not required for the studies on animals in accordance with the local legislation and institutional requirements because only commercially available established cell lines were used.

## Author contributions

SA: Conceptualization, Data curation, Formal Analysis, Funding acquisition, Investigation, Methodology, Software, Validation, Visualization, Writing – original draft, Writing – review & editing. ZK: Formal Analysis, Methodology, Writing – review & editing. MM: Formal Analysis, Investigation, Methodology, Writing – review & editing. MG-O: Formal Analysis, Methodology, Writing – review & editing, Investigation. NR: Conceptualization, Data curation, Formal Analysis, Investigation, Methodology, Resources, Supervision, Validation, Writing – review & editing. DP: Conceptualization, Data curation, Funding acquisition, Methodology, Project administration, Resources, Supervision, Validation, Writing – review & editing, Formal Analysis.
