# Supplementary material for: Self-assembling ferritin nanoplatform for the development of infectious hematopoietic necrosis virus vaccine
Source: Front Immunol. 2024 Jan 29;15:1346512. doi: 10.3389/fimmu.2024.1346512 (PMC10863052; doi:10.3389/fimmu.2024.1346512)
Supplement: Supplementary file 1 [file DataSheet_1.docx]

Supplementary Material

| **Supplementary Table 1:** The primers used for the RT-qPCR. | | | | | | | | |
| --- | --- | --- | --- | --- | --- | --- | --- | --- |
| Genes | | Acc. No | FW-Primer (5’-3’) | | Rev-Primer (5’-3’) | | Amplicon (bp) | |
| ***ifit5*** | NM_001124333 | | | GGGTAGCCTATTCCGCGTACTT | | CTGCTTTGACCGAGGCACTC | | 80 |
| ***vig1*** | NM_001124253 | | | AACGCTGGGGAGAACAGTCT | | TCCCCTCTCGGCAATCCA | | 181 |
| ***isg-15*** | NM_001124332 | | | TCAGGTGTCAATGGGAACAA | | TTTGGACCTTGGCTTTGAAC | | 205 |
| ***mx*** | NM_001171901 | | | AGCTCAAACGCCTGATGAAG | | ACCCCACTGAAACACACCTG | | 142 |
| ***ef-1 α*** | NM_001124339 | | | GATCCAGAAGGAGGTCACCA | | TTACGTTCGACCTTCCATCC | | 150 |


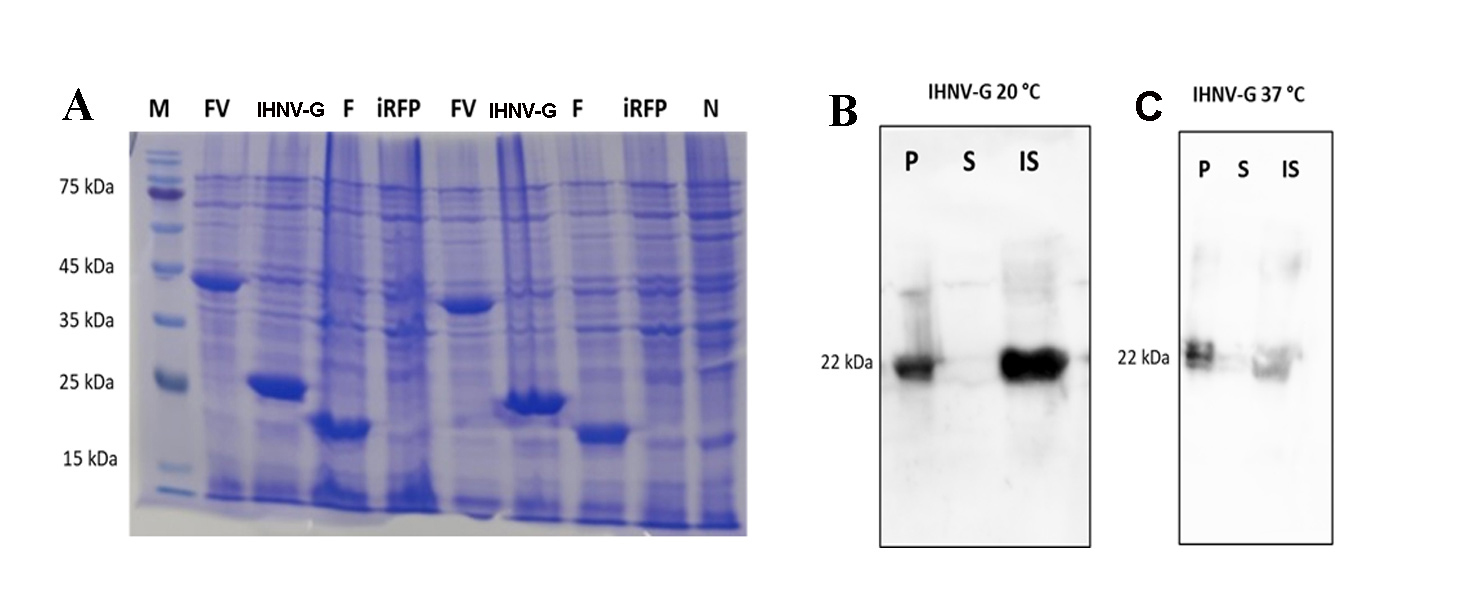


**Supplementary Figure 1.** SDS-PAGE assay (12%) for proteins induced by 1 mM IPTG (A): Lanes are M: Marker; FV: FerritVAC (42 kDa); IHNV-G (22 kDa); F: Ferritin (20 kDa); iRFP (36 kDa) and N: negative control. (B) Western blot analysis of IHNV-G (22 kDa) after IPTG induction overnight at 20 °C (B) and 3h at 37 °C (C). Total protein production (P) after IPTG induction of culture; Soluble (S) and insoluble (IS) fractions after IPTG induction and cell lysis.


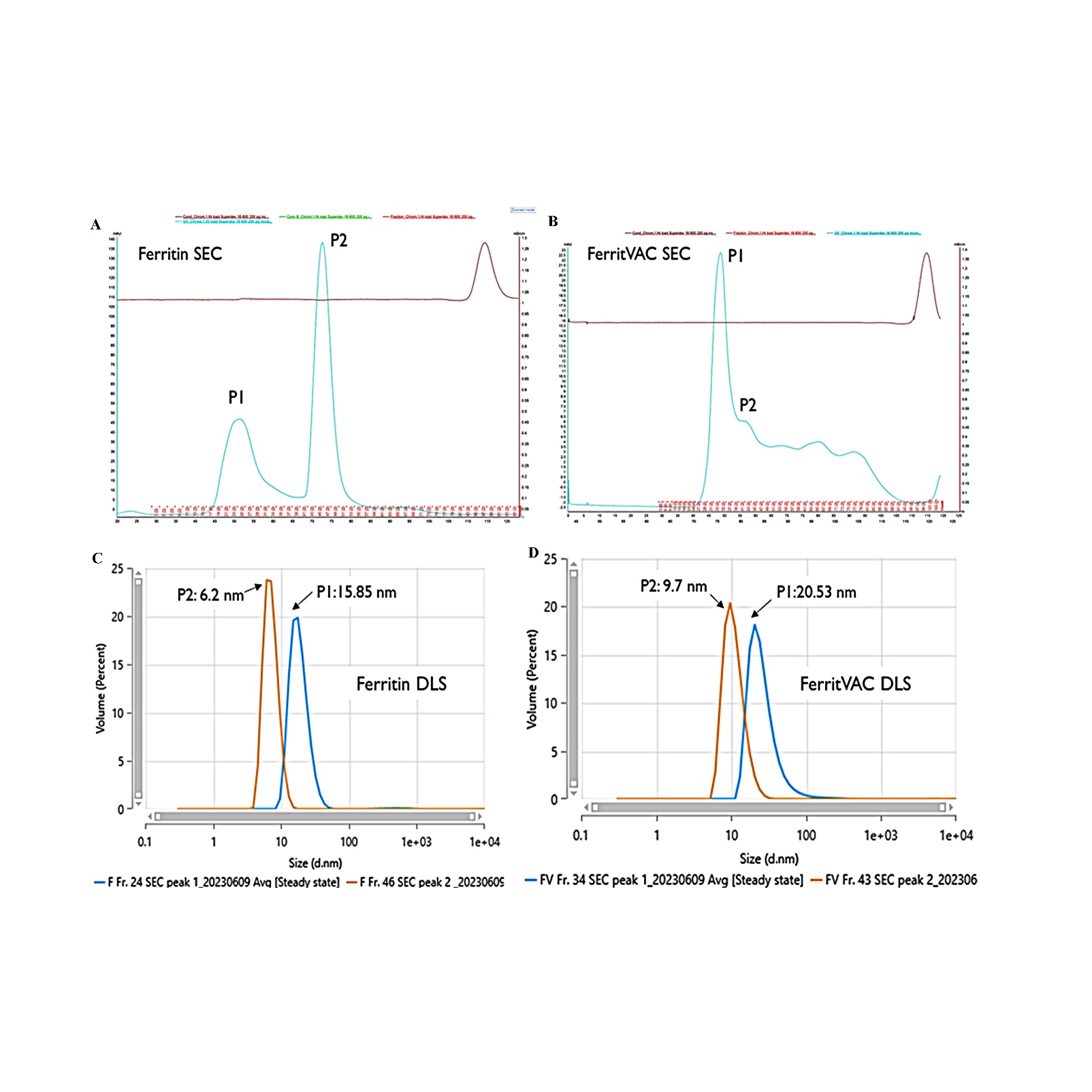


**Supplementary Figure 2.** Size exclusion chromatograms (A and B) and Dynamic Lights Scattering (C and D) showing fractions corresponding to monomers (P2) and trimers (P1) of FerritVac and Ferritin NPs.
